# Transapical transcatheter aortic valve replacement in patients with or without prior coronary artery bypass graft operation

**DOI:** 10.1186/s13019-016-0551-7

**Published:** 2016-11-29

**Authors:** Konstantinos V. Voudris, S. Chiu Wong, Ryan Kaple, Polydoros N. Kampaktsis, Andreas R. de Biasi, Jonathan S. Weiss, Richard Devereux, Karl Krieger, Luke Kim, Rajesh V. Swaminathan, Dmitriy N. Feldman, Harsimran Singh, Nikolaos J. Skubas, Robert M. Minutello, Geoffrey Bergman, Arash Salemi

**Affiliations:** 1 William Acquavella Heart Valve Center, New York-Presbyterian Hospital/Weill Cornell Medical College, 525 East 68th St., New York, NY USA; 2 Department of Cardiothoracic Surgery, New York-Presbyterian Hospital/Weill Cornell Medical College, 525 East 68th St., New York, NY USA; 3 Department of Cardiology, New York-Presbyterian Hospital/Weill Cornell Medical College, 525 East 68th St., New York, NY USA; 4 Department of Anesthesiology, New York-Presbyterian Hospital/Weill Cornell Medical College, 525 East 68th St., New York, NY USA; 5 Department of Cardiology, Duke University Medical Center and the Duke Clinical Research Institute, Durham, NC USA

**Keywords:** Aortic valve stenosis, Coronary artery bypass grafting, Transcatheter aortic valve replacement, Outcomes

## Abstract

**Background:**

Transapical approach (TA) is an established access alternative to the transfemoral technique in patients undergoing transcatheter aortic valve replacement (TAVR) for treatment of symptomatic aortic valve stenosis. The impact of prior coronary artery bypass grafting (CABG) on clinical outcomes in patients undergoing TA-TAVR is not well defined.

**Methods:**

A single center retrospective cohort analysis of 126 patients (male 41%, mean age 85.8 ± 6.1 years) who underwent TA balloon expandable TAVR (Edwards SAPIEN, SAPIEN XT or SAPIEN 3) was performed. Patients were classified as having prior CABG (*n* = 45) or no prior CABG (*n* = 81). Baseline clinical characteristics, in-hospital, 30-day, 6 months and one-year clinical outcomes were compared.

**Results:**

Compared to patients without prior CABG, CABG patients were more likely to be male (62.2 vs. 29.6%, *p* < 0.001) with a higher STS score (11.66 ± 5.47 vs. 8.99 ± 4.19, *p* = 0.003), history of myocardial infarction (55 vs. 21.1%, *p* < 0.001), implantable cardioverter defibrillator (17.8 vs. 3.7%, *p* = 0.017), left main coronary artery disease (42.2 vs. 4.9%, *p* < 0.001), and proximal left anterior descending coronary artery stenosis (57.8 vs. 16%, *p* < 0.001). They also presented with a lower left ventricular ejection fraction (%) (42.3 ± 15.3 vs. 54.3 ± 11.6, *p* < 0.01) and a larger effective valve orifice area (0.75 ± 0.20 cm^2^ vs. 0.67 ± 0.14 cm^2^, *p* = 0.025). There were no intra-procedural deaths, no differences in stroke (0 vs. 1.2%, *p* = 1.0), procedure time in hours (3.50 ± 0.80 vs. 3.26 ± 0.86, *p* = 0.127), re-intubation rate (8.9 vs. 8.6% *p* = 1.0), and renal function (highest creatinine value 1.73 ± 0.71 mg/ml vs.1.88 ± 1.15 mg/ml, *p* = 0.43). All-cause mortality at 6 months was similar in both groups (11.4, vs. 17.3% *p* = 0.44), and one-year survival was 81.8 and 77.8% respectively (*p* = 0.51). On multivariate analysis, the only factor significantly associated with one-year mortality was prior history of stroke (HR, 2.76; 95% CI, 1.06-7.17, *p* = 0.037).

**Conclusion:**

Despite the higher baseline clinical risk profile, patients with history of prior CABG undergoing TA-TAVR had comparable in-hospital, 6 months and one-year clinical outcomes to those without prior CABG.

## Background

Redo cardiac surgery is associated with an increased risk of morbidity and mortality in comparison with an initial cardiac operation [1–3]. In patients with prior coronary artery bypass grafting (CABG) and patent grafts undergoing reoperation for surgical aortic valve replacement (SAVR), in addition to technical issues related to cicatrix formation and dissection, myocardial protection and prevention of graft injuries are concerns that require careful surgical management [3, 4].

Transcatheter aortic valve replacement (TAVR) has shown good early and mid-term results in patients with severe aortic stenosis and various comorbidities or inoperable patients [5–7]. High-risk or inoperable patients with severe aortic stenosis who had previously undergone cardiac surgery represent a particularly challenging group that could benefit from a minimally invasive trans-catheter approach. Trans-apical (TA) TAVR has become an alternative access site for the treatment of high-risk patients with severe symptomatic aortic stenosis. Because of the minimally invasive nature of this approach, TA-TAVR has become particularly attractive in high-risk patients with prior cardiac surgery [8, 9]. However up to now the impact of prior CABG on the clinical outcome after TA-TAVR has yet to be defined. The purpose of the present study was to compare outcomes of patients with prior CABG undergoing TA-TAVR with those undergoing TA-TAVR as an initial procedure.

## Methods

### Patients

Between October 2010 and December 2014, 126 consecutive high-risk patients (male 41%, mean age 85.8 ± 6.1) underwent TA-TAVR. Of these, 45 patients (36%) have had prior CABG. Patients included in the study were symptomatic adults (94% had NYHA class III and IV) with severe aortic stenosis who were not candidates for SAVR because of co-morbidities*.* Severe aortic stenosis was defined by effective valve orifice area <0.8 cm^2^, mean aortic valve gradient ≥40 mmHg, or peak aortic jet velocity ≥4.0 m per second. Risk factors for cardiovascular surgery were assessed on the Society for Thoracic Surgeons (STS) calculator (STS score >10% indicating very high surgical risk). Patient data were collected prospectively during treatment using standardized forms to record demographic and clinical characteristics as well as procedural data. Pre-interventional patient screening included trans-thoracic echocardiography and coronary artery angiography for exclusion of coronary artery disease. For patients identified with coronary artery disease, a revascularization procedure was performed prior to the valve replacement. Aortic and mitral regurgitation were assessed in all relevant views using color and spectral Doppler. Transvalvular regurgitation was graded according to American Society of Echocardiography recommendations as none, trace, mild, moderate, or severe. Follow-up was obtained at 30 days, 6 months and one year based on the medical records and on physician and patient interviews. All patients had trans-thoracic echocardiography at 30 days and one-year clinical follow-up. The primary end-point of this analysis was death from any cause at 6 months and one year. Secondary safety end-points were major adverse events as defined by the Society for Thoracic Surgeons and the American College of Cardiology TVT Registry™ (https://www.ncdr.com/WebNCDR/docs/default-source/tvt-public-page-documents/tvt-registry-2_0_coderdatadictionary.pdf?sfvrsn=2) and Valve Academic Research Consortium (VARC) [10] criteria. The therapeutic options of SAVR (with CABG where applicable) or TA-TAVR were discussed extensively with all patients. The choice of treatment was made at the discretion of the heart team, consisting of cardiac surgeons and interventional cardiologists, based on individual risk assessment, patient preference and a transfemoral-first evaluation process. The study protocol was approved by the institutional review board at the medical school and individual patient consent was obtained.

### Aortic valve implantation

Procedural details of the technique have been previously described [11]. All operations were performed in a specially equipped angiography suite that fulfills the standards of a hybrid operating room. Besides standard hemodynamic monitoring, transesophageal echocardiography was routinely utilized and cardiopulmonary bypass was available in all cases.

A transverse incision approximately 8 cm in length was created in the inframammary position. This was carried through subcutaneous tissues using Bovie electrocautery. A retractor was placed and pleural adhesions were carefully dissected free. The pericardium was identified and carefully opened. Adhesions of the heart to the pericardial cavity were carefully dissected. An area approximately 2 cm superior and 2 cm lateral to the true apex was identified. Circumferential pursestring sutures were placed using 2–0 Prolene suture. Heparin was given to achieve an ACT of greater than 250 s. The apex was accessed with a needle and through the use of a Supra Core wire (Abbott Vascular, Santa Clara, CA, USA) the aortic valve was crossed. The commercially available balloon-expandable aortic valve prosthesis Edwards SAPIEN™, SAPIEN XT™ or SAPIEN 3™ was used (Edwards Lifesciences, Irvine, CA, USA). Fluoroscopy and trans-esophageal echocardiography were used to guide the catheter across the native valve and direct deployment of the stent at the level of the annulus. During a period of rapid pacing, the valve was deployed. Valve function was immediately assessed by angiographic and echocardiographic visualization. Pursestring sutures were tied and a buttressing 3–0 Prolene pledgeted suture was placed in a mattress fashion. A left lateral chest tube was inserted. The incision was closed in a standard fashion and sterile dressings were applied.

### Statistical analysis

Data are presented as frequency distributions and percentages. All continuous data are expressed as mean ± standard deviation and categorical data are reported as count (percent). Categorical variables were analyzed with the Fischer’s exact test or *X* ^2^ test and continuous variables with Student’s t-tests for means with normal distribution, and the Wilcoxon rank sum test for skewed data. Time-to-event data were summarized by frequencies and log-rank statistics. For one-year survival, Kaplan-Meier estimates were calculated and compared using the log-rank test. A univariate Cox model was used to analyze one-year survival data, with a *P* value of less than 0.10 indicating statistical significance. A multivariate Cox model with stepwise regression, adjusted for history of stroke, dyslipidemia, presence of pre-procedure atrial fibrillation, STS score, NYHA class, severity of pre-procedure aortic regurgitation and new onset atrial fibrillation was used to assess hazard ratios (HR) and 95% confidence interval (CI). A *P*-value of 0.05 indicated statistical significance. All statistics were computed with the SPSS software (SPSS 21.0 for Windows, SPSS Inc).

## Results

### Patient characteristics and co-morbidities

One hundred and twenty-six consecutive patients underwent TA-TAVR at our institution during the study period; 45 (36%) had a prior history of CABG. Table 1 summarizes the preoperative characteristics of this cohort. Compared to patients without prior CABG, CABG patients were more likely to be male (62,2 vs. 29.6%, *p* < 0.001), with higher prevalence of dyslipidemia (88.9 vs. 72.8%, *p* = 0.04) and diabetes (44.4 vs. 23.5%, *p* = 0.02), history of myocardial infarction (55 vs. 21.1%, *p* < 0.001), implantable cardioverter defibrillator (17.8 vs. 3.7%, *p* = 0.017), left main coronary artery disease (42.2 vs. 4.9%, *p* < 0.001) and proximal left anterior descending coronary artery stenosis (57.8 vs. 16%, *p* < 0.001). The mean calculated STS score was higher in the CABG group (11.66 ± 5.47 vs. 8.99 ± 4.19, *p* = 0.003). Echocardiography before valve implantation showed significantly lower left ventricular ejection fraction (%) (42.3 ± 15.3 vs. 54.3 ± 11.6, *p* < 0.01), a slightly larger effective valve orifice area (0.75 ± 0.20 cm^2^ vs. 0.67 ± 0.14 cm^2^, *p* = 0.025), a larger left ventricular diameter at end systole (4.05 ± 1.88 cm vs. 3.42 ± 1.21 cm, *p* = 0.026) and a higher prevalence of moderate/severe mitral valve regurgitation (16.0 vs. 35.6%, *p* = 0.016) in patients with prior CABG.

**Table 1:** Baseline characteristics of patients of the Prior Coronary Artery Bypass Graft Surgery and the Initial Transapical Transcatheter Aortic Valve Replacement Groups

	No CABG (*N* = 81)	CABG (*N* = 45)	*P* Value
Age	85.8 ± 6.1	82.5 ± 7.4	0.008
Male, (%)	24 (29.6)	28 (62.2)	<0.001
BMI	25.58 ± 4.84	25.41 ± 4.70	0.853
STS Score	8.99 ± 4.19	11.66 ± 5.47	0.003
NYHA III or IV	56 (69.1)	34 (75.6)	0.539
Permanent PM	9 (11.1)	10 (22.2)	0.120
ICD	3 (3.7)	8 (17.8)	0.017
Atrial Fibrillation	27 (33.3)	20 (44.4)	0.251
Previous MI	16 (21.1)	22 (55)	<0.001
Prior PCI	41 (50.6)	20 (44.4)	0.578
Stroke	8 (9.9)	7 (15.6)	0.395
Transient Ischemic Attack	6 (7.4)	6 (13.3)	0.346
PAD	25 (30.9)	20 (44.4)	0.174
Smoking History	52 (64.2)	30 (66.7)	0.847
Hypertension	69 (85.2)	42 (93.3)	0.253
Dyslipidemia	59 (72.8)	40 (88.9)	0.042
Diabetes	19 (23.5)	20 (44.4)	0.017
Oral Medications only	9 (11.1)	11 (24.4)	0.073
Insulin only	5 (6.2)	5 (11.1)	0.328
Immunocompromise Present	12 (14.8)	1 (2.2)	0.031
Left Main Stenosis > 50%	4 (4.9)	19 (42.2)	<0.001
Proximal LAD > 70% stenosis	13 (16.0)	26 (57.8)	<0.001
Renal Dialysis	3 (3.7)	1 (2.2)	1.0
Creatinine mg/dl	1.28 ± 0.65	1.32 ± 0.48	0.686
Echocardiography			
Aortic Valve Area, cm^2^	0.67 ± 0.14	0.75 ± 0.20	0.025
LVEF (%)	54.3 ± 11.6	42.3 ± 15.3	<0.001
Aortic Valve Mean Gradient, mm Hg	47.7 ± 12.5	44.1 ± 11.9	0.126
Aortic Valve Peak Gradient, mm Hg	82.5 ± 22.4	75.8 ± 18.6	0.091
Aortic Valve Regurgitation Moderate/Severe	1 (1.2)	1 (2.2)	1.0
Mitral Valve Regurgitation Moderate/Severe	13 (16.0)	16 (35.6)	0.016
LVIDs, cm	3.42 ± 1.21	4.05 ± 1.88	0.026
LVIDd, cm	4.96 ± 1.14	5.30 ± 1.91	0.212

Results are number (%) or mean ± standard deviation

*CABG* Coronary artery bypass graft, *BMI* Body mass index, *NYHA* New York Heart Association classification, *STS* Society of Thoracic Surgeons, *PM* Pacemaker, *ICD* Implantable cardioverter defibrillator, *MI* Myocardial infarction, *PCI* Percutaneous coronary intervention, *PAD* Peripheral arterial disease, *LAD* Left anterior descending, *LVEF* Left ventricular ejection fraction, *LVIDs* Left ventricular diameter at end systole, *LVIDd* Left ventricular diameter at end diastole

### Procedural data and in-hospital outcomes

Procedural data for the study cohort are shown in Table 2. All patients received a balloon-expandable Edwards SAPIEN™, Edwards SAPIEN XT™ or Edwards SAPIEN 3™ aortic valve prosthesis; a 23 mm bioprosthesis was more frequently used in the non CABG group (66.3 vs. 35.6%), whereas a 26 mm bioprosthesis was more frequently used in the CABG group (62.2 vs. 32.5%) (*p* = 0.002). Mean surgical time in hours was slightly higher, but not significantly different, in prior CABG patients (3.50 ± 0.80 vs. 3.26 ± 0.86, *p* = 0.127). There were no conversions to open heart surgery, no aborted procedures, and no intra-operative deaths. In two patients with prior CABG cardiopulmonary bypass through femoro-femoral cannulation was used for persistent hypotension (4.4% vs. 0, *p* = 0.126). In-hospital length of stay, stroke, myocardial infarction, duration of intubation, re-intubation, renal function after the procedure, conduction abnormalities (new left bundle brunch block or right bundle brunch block) and VARC defined bleeding and vascular complications were similar between the two groups. Post-procedure new onset atrial fibrillation was significantly lower in the CABG group (11.1 vs. 30.9%, *p* = 0.016). Permanent pacemaker implantation was required in 4.9% of patients without and 6.7%, with prior CABG (*p* = 0.7).

**Table 2:** Procedural characteristics and in-hospital outcomes

	No CABG (*n* = 81)	CABG (*n* = 45)	*p* Value
Procedural Data			
Valve size			
23	53 (66.3)	16 (35.6)	0.002
26	26 (32.5)	28 (62.2)	
29	1 (1.3)	1 (2.2)	
Procedure Time (Hours)	3.26 ± 0.86	3.50 ± 0.80	0.127
Cardiopulmonary Bypass	0	2 (4.4)	0.126
Outcomes			
Stroke	1 (1.2)	0	1.0
MI	0	0	
New Renal Failure requiring Hemodialysis	5 (6.2)	1 (2.2)	0.420
Respiratory Distress	19 (23.5)	12 (26.7)	0.829
Re-Intubation	7 (8.6)	4 (8.9)	1.0
Coronary Obstruction	0	0	
In Hospital Creatinine, (highest value, mg/dl)	1.88 ± 1.15	1.73 ± 0.71	0.430
Creatinine at discharge (mg/dl)	1.33 ± 0.67 (*N* = 76)	1.40 ± 0.57	0.560
Hospitalization Duration	10.48 ± 6.7	9.64 ± 7.9	0.531
Arrhythmias			
Conduction Disturbance	12 (14.8)	6 (13.3)	1.0
New LBBB	10 (12.3)	6 (13.3)	1.0
New RBBB	2 (2.5)	0	0.537
New Atrial Fibrillation	25 (30.9)	5 (11.1)	0.016
New Other Significant Arrhythmia	13 (16.0)	5 (11.1)	0.597
Permanent Pacemaker Implantation	4 (4.9)	3 (6.7)	0.700
Bleeding			
Life Threatening Bleeding^a^	4 (4.9)	1 (2.2)	0.654
Major Bleeding^a^	28 (34.6)	15 (33.3)	1.0
Gastrointestinal Bleeding	4 (4.9)	0	0.296
Genitourinary Bleeding	4 (4.9)	3 (6.7)	0.700
Transapical approach related	4 (4.9)	2 (4.4)	1.0
PRBC Transfusion	48 (59.3)	29 (64.4)	0.703
# Units transfused	2.2 ± 2.0	2.1 ± 1.4	0.947
Vascular Complications			
Major Vascular Complication	1 (1.2)	0	1.0
Minor Vascular Complication	1 (1.2)	0	1.0
Annular Dissection	0	0	
Requirement of CT surgery	0	2 (4.4)	0.126
Requirement of Vascular Surgery	0	0	

Results are number (%) or mean ± standard deviation

*MI* Myocardial infarction, *LBBB* Left bundle branch block, *RBBB* Right bundle branch block, *PRBC* Packed red blood cells, *CT* Cardiothoracic surgery

^a^Defined using VARC criteria^10^

### One-year clinical outcomes

**Fig. 1:**
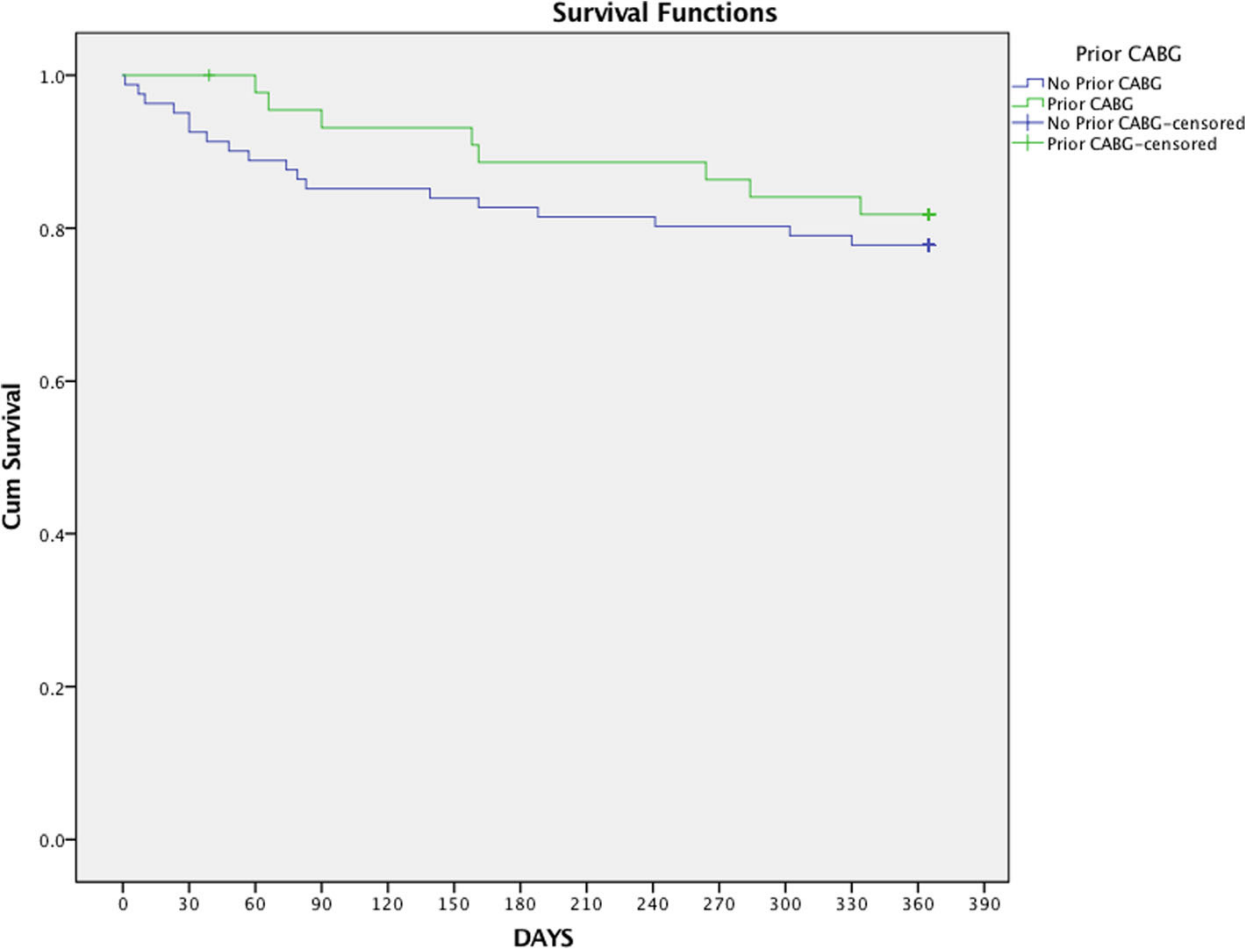
Time-to-event curves for cumulative survival. Events were calculated with the use of Kaplan-Meier methods. (Previous CABG: green line; No CABG: blue line) *p* = 0.509

All but one patients had clinical follow up at 6 months and one year. All-cause mortality at six months (11.4 vs. 17.3%, *p* = 0.44) and one year (18.2 vs. 22.2%, *p* = 0.65) were similar in patients with and without prior CABG. Similar results were observed with cardiovascular mortality (Table 3). Survival at one year was 81.8% for patients with and 77.8% for patients without prior CABG (*p* = 0.51) (Fig. 1). Improvements in echocardiographic parameters were marked and sustained both at 30 days and at one-year relative to pre-procedure values (Table 4). There were no cases of recurrent aortic stenosis or moderate/severe aortic insufficiency in either group of patients. At 30 days, the effective aortic valve area was 1.64 ± 0.25 cm^2^ and 1.62 ± 0.31 cm^2^ (*p* = 0.66) in patients with and without prior CABG and mean gradient was 9.7 ± 3.3 mm Hg and 10.2 ± 3.9 mm Hg (*p* = 0.54) respectively; at one-year effective aortic valve area was 1.82 ± 0.35 cm^2^ and 1.66 ± 0.40 cm^2^ (*p* = 0.08) with a mean gradient of 10.0 ± 4.0 mm Hg and 11.6 ± 6.4 mm Hg (*p* = 0.23) respectively. Patients with prior CABG maintained a lower left ventricular ejection fraction (47.2% ± 14.3% vs. 57.1% ± 9.6%, *p* < 0.001 and 43.9% ± 16.1%, vs. 60.0% ± 8.1% *p* < 0.001) at 30 days and one year respectively; however, the changes (Δ) on left ventricular ejection fraction pre and post procedure (at 30 days and one year) were not statistically different in both groups of patients. Post-operative incidence of moderate/severe mitral valve regurgitation was comparable between the two groups at 30 days and one-year (12.2 vs 20.0%, *p* = 0.295 and 12.5 vs 14.2%, *p* = 1.0, respectively). On multivariate analysis, the only factor significantly associated with one-year mortality was prior history of stroke (HR, 2.76; 95% CI, 1.06-7.17, *p* = 0.037). Neither prior CABG nor new onset atrial fibrillation were significantly associated with 1-year mortality (HR, 0.76; 95% CI, 0.33-1.74, *p* = 0.51 and HR, 1.76; 95% CI, 0.78-3.95, *p* = 0.17 respectively).

**Table 3:** Mortality at clinical follow-up

	No CABG (*n* = 81)	CABG (*n* = 44)	*p* Value
At 6 months			
All-Cause Mortality	14 (17.3)	5 (11.4)	0.444
Cardiovascular Mortality	5 (6.2)	2 (4.5)	
At 1 year			
All-Cause Mortality	18 (22.2)	8 (18.2)	0.651
Cardiovascular Mortality	7 (8.6)	3 (6.8)	

Data expressed as n (%)

**Table 4:** Echocardiographic results at clinical follow-up

	No CABG (*n* = 75)	CABG (*n* = 45)	*p* Value
At 30 Days			
Aortic Valve Area, cm^2^	1.62 ± 0.31	1.64 ± 0.25	0.655
LVEF, %	57.1 ± 9.6	47.2 ± 14.3	<0.001
Aortic Valve Mean Gradient, mm Hg	10.2 ± 3.9	9.7 ± 3.3	0.534
Aortic Valve Peak Gradient, mm Hg	20.1 ± 7.0	19.9 ± 6.9	0.923
LVIDs, cm	3.59 ± 0.66	4.30 ± 1.0	<0.001
LVIDd, cm	5.13 ± 0.64	5.64 ± 0.82	<0.001
Δ LVEF, %	2.6 ± 9.6	3.6 ± 7.7	0.560
Δ LVIDs, cm	−0.06 ± 0.51	−0.27 ± 0.46	0.028
Δ LVIDd, cm	0.02 ± 0.52	−0.15 ± 0.61	0.119
AV Regurgitation Moderate/Severe	0	0	
MV Regurgitation Moderate/Severe	9 (12.2)	9 (20.0)	0.295
At One Year	No CABG (*n* = 48)	CABG (*n* = 28)	
Aortic Valve Area, cm^2^	1.66 ± 0.40	1.82 ± 0.35	0.079
LVEF, %	60.0 ± 8.1	43.9 ± 16.1	<0.001
Aortic Valve Mean Gradient, mm Hg	11.6 ± 6.4	10.0 ± 4.0	0.234
Aortic Valve Peak Gradient, mm Hg	22.8 ± 12.3	19.0 ± 5.9	0.137
LVIDs	3.39 ± 0.46	4.55 ± 1.37	<0.001
LVIDd, cm	5.02 ± 0.49	5.80 ± 1.09	<0.001
Δ LVEF, %	3.5 ± 7.9	4.9 ± 7.7	0.470
Δ LVIDs, cm	−0.21 ± 0.55	−0.18 ± 0.63	0.868
Δ LVIDd, cm	−0.07 ± 0.54	−0.02 ± 0.66	0.758
AV Regurgitation Moderate/Severe	0	0	
MV Regurgitation Moderate/Severe	6 (12.5)	4 (14.2)	1.0

*LV EF* Left ventricular ejection fraction, *LVIDs* Left ventricular diameter at end systole, *LVIDd* Left ventricular diameter at end diastole. Δ calculated from baseline pre procedure measurements, *AV* Aortic valve, *MV* Mitral valve

## Discussion

This study demonstrates that prior CABG in patients undergoing TA-TAVR does not impair clinical outcome. Patients with CABG presented herein had a clearly elevated risk profile as expressed by a higher STS risk score, an increased prevalence of dyslipidemia, diabetes, previous myocardial infarction and significant left main stenosis, and a lower left ventricular ejection fraction. Despite the high risk profile, procedural aspects, post-procedural complication rates, and one-year clinical outcome, were comparable between the two groups; one-year survival was 81.8 and 77.8% for patients with and without prior CABG respectively (*p* = 0.509). Interestingly, patients with prior CABG exhibited a smaller incidence of new onset atrial fibrillation (*p* = 0.016). However, on multivariate analysis new onset atrial fibrillation did not significantly increase the hazard for one-year mortality. Conduction abnormalities and permanent pacemaker placement rate were comparable between the two groups. Echocardiography measurements of the aortic valve were similar both at 30 days and at one year and changes on left ventricular ejection fraction at follow up were not statistically different in both groups of patients. The only predictor for mortality on multivariate analysis was history of stroke.

TAVR has been documented to be safe and effective in inoperable patients with severe aortic stenosis and has also emerged as an alternative to open SAVR in high-risk patients with severe aortic stenosis [5, 6]. Patients undergoing cardiac re-operation consist a high-risk subgroup [12]. Despite previous reports showing that SAVR can be performed safely and with excellent outcomes in patients with prior CABG [13, 14], the risk of mortality and morbidity in this subgroup is higher. Repeat sternotomy pose the risk of coronary graft injury during re-entry to the mediastinum and difficulty in myocardial protection as well as potential traumatic injury and hemorrhage [3, 12]. Moreover, multiple co-morbidities render this population as a surgically challenging group. Thus, TAVR has been suggested as a viable option in these patients. The proportion of TAVR patients with prior CABG has been reported to range from 25.5 to 42% [15]. TAVR patients with a history of prior cardiac surgery have documented to be younger and have more co-morbidities, such as diabetes, porcelain aorta, peripheral vascular disease and poor left ventricular ejection fraction compared with patients without previous cardiac operation [2, 15].

Several studies have shown the feasibility of TAVR in patients with prior CABG regardless of the access site. On multivariate analysis of PARTNER I trial, where high-risk patients with severe aortic stenosis were randomized to SAVR or TAVR, prior CABG was shown to have no impact on prognosis after TAVR [16]. In a subgroup analysis of the same trial no differences between patients with previous cardiac operation following TAVR vs SAVR in terms of operative outcomes were found; however, a trend towards greater all-cause mortality was seen in the TAVR group at 2 years [17]. Similarly, in a recent report from the European SOURCE XT registry, which included 2.688 patients who underwent TAVR with the SAPIEN XT valve, prior CABG was not associated with one year mortality [18]. These findings are in accordance with data from FRANCE 2 Registry in which 30-day and one-year mortality from all causes did not differ according to history of CABG [19]. Moreover, there were no significant differences in VARC complications (myocardial infarction, stroke or vascular and bleeding complications). On multivariate analysis, CABG was not associated with greater one year post-TAVR mortality. In their study of 201 patients, 140 (70%) of whom underwent TAVR through trans-arterial approach and 61 patients through TA approach, Ducrocq et al. reported similar mortality rates in TAVR patients irrespective of prior CABG history and concluded that TAVR is an attractive option in high risk population with symptomatic severe aortic stenosis and previous heart surgery [8].

Currently, only a few studies have reported on prognosis after TA-TAVR in relation to prior CABG and the exact impact of previous cardiac operation on mortality after TA-TAVR is not well established. In a retrospective study of 566 patients, 110 (19.4%) with a history of prior CABG, who underwent TA-TAVR from the Italian Registry all-cause and cardiovascular mortality at 30 days and survival at one and two years were similar between patients with and without prior CABG [2]. Additionally, there were no significant differences between the two groups in terms of post-procedure complications. In line with the above study, no differences in the short-term and long-term mortality were documented between propensity-matched subgroups of 45 TAVR patients with and 45 TAVR patients without prior CABG in a study by Papadopoulos et al. [20]. Echocardiography at 4 years showed no differences in left ventricular ejection fraction, effective orifice valve area and mean trans-valvular gradient between the two groups in this study. The findings of the above studies were also comparable with reports by Drews et al. [21], and Walther et al. [22] and indicate that prior CABG does not have a significant impact morbidity and mortality in patients undergoing TA-TAVR.

Our results are in accordance with the existing literature. Mortality at six months and one year follow up were similar between the two groups, while echocardiography data at 30 days and one year with respect to aortic valve orifice, and trans-valvular gradient were not different. Moderate/severe mitral valve regurgitation frequency at 30 days and one-year after valve implantation was similar between the two groups and improved from the pre procedure one. Toggweiler et al. in their study on patients with moderate and severe mitral valve regurgitation had previously reported an improvement in mitral regurgitation in 55% of patients with moderate or severe MR after TAVR at one-year follow up [23]. Additionally, a lower incidence of new onset atrial fibrillation was observed post-procedure in patients with prior CABG (11.1 vs. 30.9%, *p* = 0.016). Higher prevalence of permanent pacemaker and implantable cardioverter defibrillator observed preoperatively in the CABG population could account for this finding. Yet, when adjusted for presence of permanent pacemaker and implantable cardioverter defibrillator, incidence of atrial fibrillation remained significantly higher in the non CABG population (*p* = 0.024). Furthermore, on multivariate analysis new onset atrial fibrillation was not predictor for all-cause mortality at one year. Cardiac surgery has been shown to increase susceptibility to atrial fibrillation in previous studies [24, 25]. In our study the prevalence of pre procedure atrial fibrillation was not statistically different in patients with prior CABG (44.4 vs. 33.3%, *p* = 0.251). The deleterious impact of atrial fibrillation on TAVR patients was recently underscored in a sub-analysis from PARTNER trial [26]. Patients who converted from sinus rhythm to atrial fibrillation by discharge had the highest rates of all-cause mortality at 30 days (adjusted HR = 3.41; *p* = 0.0002) and over 2-fold difference at 1 year (adjusted HR = 2.14; *p* < 0.0001). The presence of atrial fibrillation on baseline or discharge ECG was a predictor of one-year mortality. Similarly, in the FRANCE 2 Registry pre-existing and new-onset atrial fibrillation are both associated with higher mortality and morbidity after TAVR [27]. In another study new onset atrial fibrillation was detected in 21% of patients; 50% of all the episodes occurred in the initial 24 h after the procedure [28]. TA approach was observed to an important predictor of new onset atrial fibrillation and is associated with a prolongation of intensive care unit and hospital stay. In a recent meta-analysis of twenty-six studies, 14,078 patients undergoing TAVR, of whom 33.4% had pre-existing atrial fibrillation and 17.5% had new onset atrial fibrillation, were assessed for early and long-term all-cause mortality, cardiovascular mortality and cerebrovascular events. New onset atrial fibrillation patients showed similar short- and long-term all-cause mortality when compared to patients in sinus rhythm, whereas a non-significant increase in the incidence of cerebrovascular events was observed at long-term follow-up [29]. On the same line in another study new onset atrial fibrillation was not associated with higher stroke or mortality rates at 30 days or one year of follow-up [30]. In multivariate analysis, atrial fibrillation pre-procedure but not new onset atrial fibrillation was a significant predictor of mortality throughout the follow-up period (HR 2.2, *p* = 0.003 at 30 days, and HR 1.5, *p* = 0.39, respectively).

From the technical point of view, procedure duration in hours, life threatening bleeding and units of blood transfused were not different between patients with and without prior CABG supporting the concept that chest reentry during TA-TAVR does not represent a substantial surgical challenge. Increased incidence of major bleeding was observed in both groups, a finding related to a combination of low baseline hemoglobin with modest peri-procedural blood loss on a high risk population. An identifiable source of bleeding was present in only a few cases and no statistical significant difference in the incidence was observed between the two groups. Our results show that adhesions and scar tissue in the apical area were not limiting factors for chest re-entry through apical access. In fact, the presence of adhesions may actually prevent myocardial tissue trauma and hemorrhage after valve implantation and apical closure, as shown by the lack of peri-operative myocardial infarction in our study patients. The potential role of patent grafts on minimizing the consequences of coronary ostia obstruction by calcium displacement or debris embolization of the coronary arteries following prosthetic valve deployment is a possible explanation for the above findings as well.

Permanent neurologic defects remain a concern in high-risk patients undergoing cardiac surgery. Stroke rate in our study, was 1.2% for patients without and 0% for patients with prior CABG undergoing TA-TAVR and were in accordance with previously published data after TA-TAVR in patients with and without prior CABG [8, 9, 31, 32]. Furthermore, history of stroke was the only predictor of one year mortality on multivariate analysis. This improvement in the prevalence of stroke certainly represents a powerful advantage of TA-TAVR in high risk patients.

### Limitations

The present study does have some limitations. This is a retrospective report from a single site and with that comes unavoidable selection and institutional biases. Larger randomized trials are required to document definitive results. Patients included in the study were treated with either the first (Edwards SAPIEN™), second (Edwards SAPIEN XT™) or third generation (Edwards SAPIEN 3™) balloon-expandable valve. This may influence the short-term and long-term clinical outcome.

## Conclusions

TA TAVR in patients with prior CABG appears to be safe and can be carried out with similar results in terms of mortality compared with patients undergoing TAVR as their first cardiac operation. TA-TAVR represents an alternative treatment option to redo SAVR in this high risk population. The decision regarding the final treatment approach should be individualized on each single patient after taking into consideration age, the associated co-morbidities and the need for revascularization.

## Abbreviations

CABG: Coronary artery bypass grafting
CI: Confidence interval
HR: Hazard ratios
NYHA: New York Heart Association classification
SAVR: Surgical aortic valve replacement
STS: Society for Thoracic Surgeons
TA: Transapical approach
TAVR: Transcatheter aortic valve replacement
VARC: Valve Academic Research Consortium
